# Infratentorial Relapsing Neuroglial Tumors in Adults: Management and Unsolved Issues—A Systematic Review

**DOI:** 10.3390/brainsci14030286

**Published:** 2024-03-18

**Authors:** Lara Brunasso, Chiara Avallone, Ada Maria Florena, Giovanni Grasso

**Affiliations:** 1 Neurosurgical Clinic, AOUP (Azienda Ospedaliera Universitaria Policlinico) “Paolo Giaccone”, Post Graduate Residency Program in Neurologi Surgery, Department of Biomedicine Neurosciences and Advanced Diagnostics, School of Medicine, University of Palermo, Via del Vespro 127, 90127 Palermo, Italy; lara.brunasso@community.unipa.it (L.B.); chiara.avallone@community.unipa.it (C.A.); 2 Department of Health Promotion Sciences, Maternal and Infant Care, Internal Medicine and Medical Specialties (PROMISE), University of Palermo, 90127 Palermo, Italy; adamaria.florena@unipa.it

**Keywords:** ganglioglioma, glioneuronal, brain tumor, infratentorial

## Abstract

(1) Background: Gangliogliomas are rare tumors accounting for about 0.4% of all central nervous system tumors. They are usually located in the temporal lobes of children and young adults, though such tumors in the infratentorial region and adult-age patients rarely reported. (2) Methods: A systematic review on ganglioglioma with infratentorial location in the adult population was conducted in accordance with the PRISMA guidelines. A total of 275 articles were found, and 23 were included. Demographic data, the location and histology of the lesion, pre-operative neurological status, the type of surgery, recurrence, radiotherapy/chemotherapy adjuvant treatments, neurological outcomes and follow-up information were collected. We also presented an illustrative case. (3) Results: A total of 27 patients were included. In 51%, the location was the cerebellum; in 40%, it was the fourth ventricle; in 11%, it was brainstem; and in 4%, it was the cerebellopontine angle. STR was performed in 44%, GTR in 26% and biopsy in 15% of the cases. Adjuvant radiotherapy was found in 22% of cases. Disease recurrence occurred in 15% of patients between 1 and 12 months after surgery with a diagnosis of high-grade ganglioglioma, while in six cases, no disease recurrence was documented. (4) Conclusions: Infratentorial glioneuronal tumors are rare findings in the adult population. Histopathological characterization does not seem to fully reflect their true behavior. Future studies are warranted for better characterizing histopathological findings and treatment.

## 1. Introduction

Glioneuronal tumors are uncommon primary central nervous system (CNS) tumors occurring during childhood and young adulthood with an estimated incidence of 0.4% of all CNS tumors, consisting of a mixture of well-differentiated neoplastic neural and glial components [1]. Ganglioma and Dysembryoplastic neuroepithelial tumors (DNET) represent the majority of this glioneuronal group, and descriptions of many other newly classified lesions (MVNT, DGONC, MGT and others) are limited to case reports and small case series [2]. According to the previous 2016 WHO classification, these tumors were classified into three grades: grade I (benign or low-grade tumors), grade II (atypical tumors) and grade III (anaplastic tumors) [1]. In the WHO CNS5 revision published in 2021, all tumors with a neuronal component remain grouped together, and three new types have been added: DGONC (provisional) tumor, myxoid glioneuronal tumor and multinodular and vacuolating neuronal tumor [3]. Typically described as well-circumscribed and slow-growing lesions, glioneuronal tumors are mostly supratentorial and temporally located [4]. Large-sized tumors may be reached before clinical recognition, and an age at diagnosis greater than 30 years old represents a rare clinical scenario [5]. While supratentorial neuroglial tumors are infrequent findings in adult populations, infratentorial, brainstem and intraventricular location are exceedingly rare [6].

Although minimal risk for an aggressive clinical course is described, the glial component may present infiltrative biological behavior into adjacent structures, worsening the prognosis [7]. The presence of microvascular proliferation, hypercellularity, the absence of oligodendrocytes, chronic inflammation, BRAFV600E mutation and methylguanine methyltransferase expression have been linked to an increased risk of recurrence in the pediatric population with diagnosed grade I ganglioglioma [8]. Despite this, detailed information about tumor behavior and the best treatments is still lacking. This systematic review summarizes the significant findings about this rare type of cerebral tumor. We also report the description of an unusual clinical case.

## 2. Materials and Methods

This systematic review was conducted according to the Preferred Reporting Items for Systematic Reviews and Meta-Analyses (PRISMA) guidelines [9], but it was not pre-registered. We performed a broad systematic literature search in the PubMed/Medline electronical database for all studies. We examined all studies published up to September 2023, without backward limits. MeSH terms used were “Ganglioglioma AND fourth ventricle”, “Ganglioglioma AND cerebellum”, “Ganglioglioma AND infratentorial” and “Ganglioglioma AND posterior fossa”. To avoid the potential omission of relevant studies, we manually screened the reference lists of articles included. Duplicate papers were eliminated using Microsoft Excel 16.37 (Redmond, WA, USA).

The research strategy initially relied on title and abstract analysis. An article’s full text was retrieved for further investigation if the title and abstract met the inclusion criteria. The data collection process was conducted without using any automated tools. No automatic tools were used in the screening and selection phases. Extensive inclusion criteria were employed due to the inadequate quantity of studies on this topic. The inclusion criteria of this study were as follows: (1) articles focusing on ganglioglioma in posterior fossa, fourth ventricle, cerebellum or infratentorial locations; (2) patients older than 18 year old; (3) papers written in the English language. The exclusion criteria were as follows: (1) papers written in a language other than English; (2) preclinical studies; (3) pediatric populations; (4) review articles.

Risk of bias assessments and study quality were conducted using the Newcastle–Ottawa Scale (NOS). The stars rating system has evaluated three categories: selection, comparability and outcome. The scores of NOS ranged from 0 stars (lowest score) to 9 stars (highest score). A study with a NOS score higher than 5 was recognized as a high-quality study [10].

Data were extracted by retrieving essential information including the author, the country, the year of publication, the number of patients examined, patient demographics (age and sex), the location and histology of the lesion, pre-operative neurological status, surgical procedure, recurrence, radiotherapy/chemotherapy, neurological outcomes and follow-up.

## 3. Results

### 3.1. Data Selection and Studies General Features

**Figure 1:**
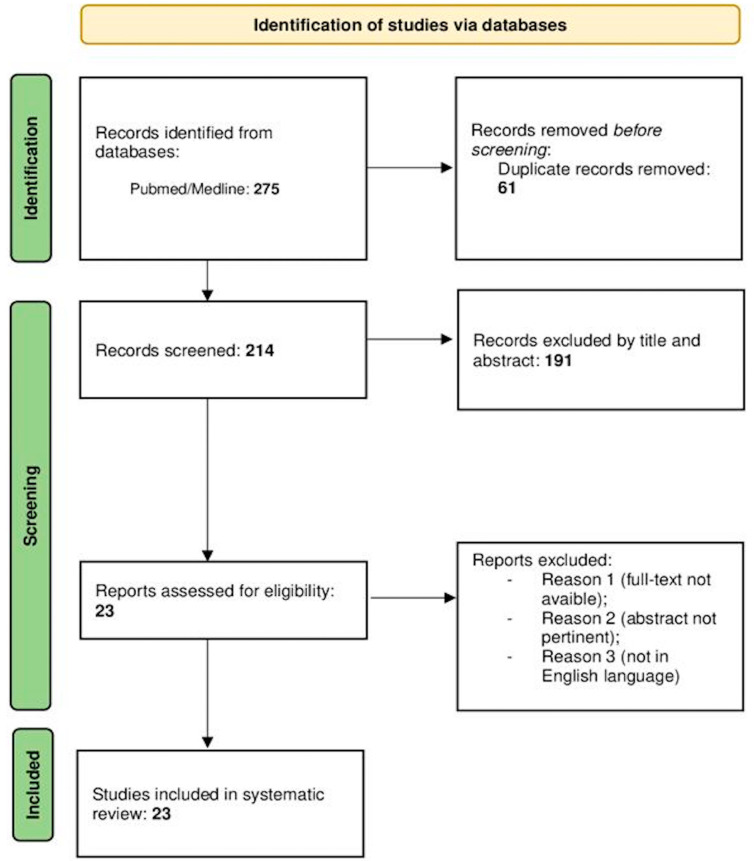
Flow diagram of the results of this systematic review according to the PRISMA guidelines.

A total of 275 articles were collected. After removing the duplicates (61), 214 articles were reviewed. Of these, 190 were excluded by title and abstract. The literature search yielded a total of 23 eligible articles for data extraction. The selected articles were published between 1966 and 2022, and not all data were available for some older papers (Figure 1).

Twelve female and fifteen male patients were identified with a median age of 36 years old (DS 32 +/− 16). Cerebellum was the most common tumor site (51%), followed by the fourth ventricle (40%), brainstem (11%) and cerebellopontine angle CPA (4%). The most common presenting symptoms were headache (51%), gait disturbances and weakness (26%), vertigo (15%), nausea (7%), ataxia (7%), vomiting (4%), intracranial hypertension (4%), syncope (3%), diplopia (4%), hemiparesis (4%), hypoventilation and apnea (4%) and slurred speech (4%). The histological diagnoses reported were ganglioglioma (22%), anaplastic ganglioglioma (11%), rosette-forming glioneuronal tumor (RGNT) (18%), ganglioblastoma (7%), gangliocytoma (4%) and xanthoastrocytoma–pleomorphic ganglioglioma combined (4%). The type of surgery and the use of adjuvant therapies were also investigated. Subtotal resection (STR) was performed in 44% of the cases, gross total resection (GTR) in 26% of the cases, simple biopsy and partial removal in 15% of the cases. Adjuvant chemotherapy was reported in 7% of the cases after STR for a cerebellar anaplastic ganglioglioma [11] and a ganglioglioma of the fourth ventricle [9]. Adjuvant radiotherapy was found in 22% of the cases and specifically after STR of anaplastic ganglioglioma of the fourth ventricle [9], anaplastic ganglioglioma of the cerebellum [11], cerebellar ganglioblastoma [12], CPA ganglioblastoma [13] and cerebellar xanthoastrocytoma–pleomorphic ganglioglioma [14]. Finally, in 15% of the cases, disease recurrence occurred between 1 and 12 months after surgery (with a mean period of 11 months). All collected characteristics included in the present systematic review are reported in Table 1.

### 3.2. Illustrative Case

**Figure 2:**
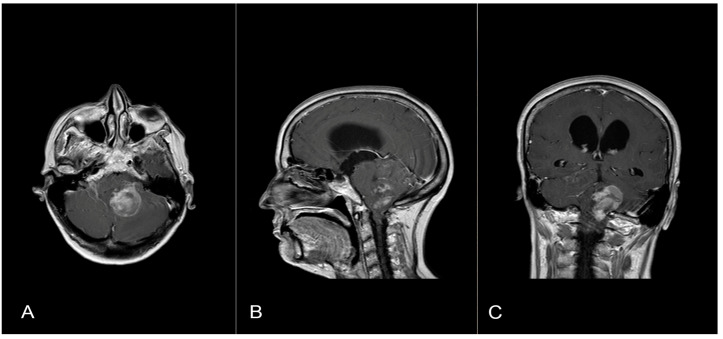
Axial (**A**), sagittal (**B**) and coronal (**C**) T1 contrast-enhanced weighted brain MRI shows a lesion in the left cerebellar hemisphere involving the middle-lower cerebellar pedicle surrounded by perilesional edema and tri-ventricular obstructive hydrocephalus for compression of the IV ventricle.

**Figure 3:**
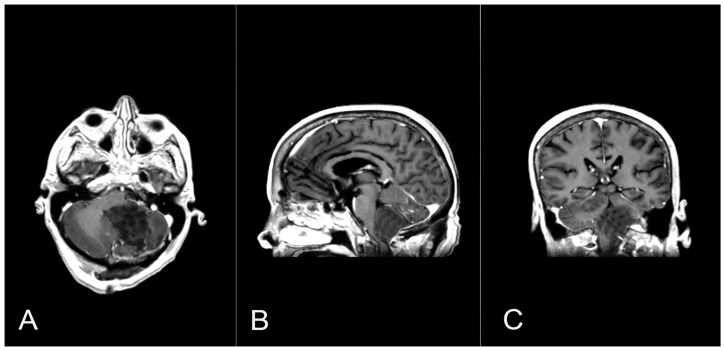
Axial (**A**), sagittal (**B**) and coronal (**C**) postoperative T1 contrast-enhanced brain MRI shows the left suboccipital craniectomy accomplished and the GTR exeresis of the lesion.

A 58-year-old woman presented with nausea, headache and gait instability. A brain contrast-enhanced magnetic resonance imaging (MRI) device documented an expansible lesion in the left cerebellar hemisphere involving the middle-lower cerebellar pedicle surrounded by perilesional edema (measures: transverse diameter: 3.2 cm; sagittal diameter: 3 cm). Compression of the IV ventricle and cerebellar tonsils herniation in the foramen magnum were present. Also, tri-ventricular obstructive hydrocephalus was present (Figure 2). The patient underwent ventricular shunt and microsurgical exeresis of the lesion via left suboccipital craniectomy (Figure 3). The histopathological examination documented a grade I glioneuronal lesion. After the first operation, the patient underwent adjuvant radiotherapy treatment with a total of 54 Gy in 27 sessions, with daily fractionation of 2 Gy/day. Clinical and radiological follow-up was then recommended.

**Figure 4:**
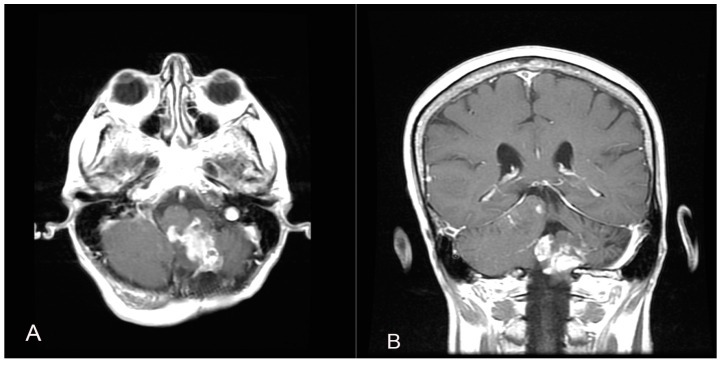
A follow-up contrast-enhanced brain MRI two years after the first surgical intervention shows the presence of a recurrence in the left cerebellar area with inhomogeneous enhancement (**A**,**B**).

**Figure 5:**
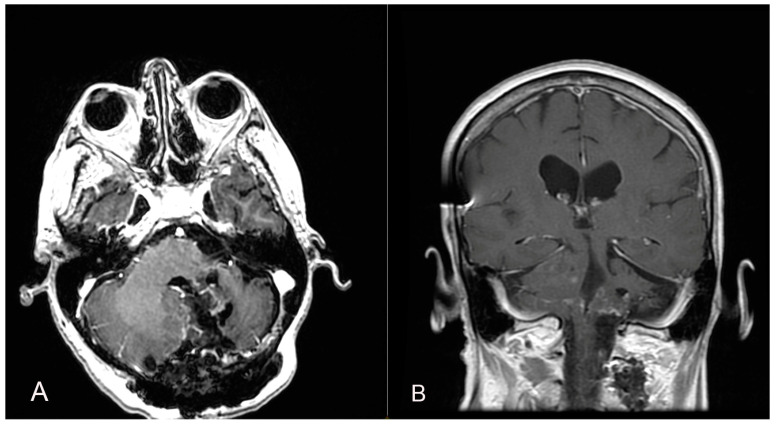
A second surgery was performed through a telo-velar approach, and postoperative contrast-enhanced brain MRI (**A**,**B**) is shown. The lesion intraoperatively appeared to be a calcified mass tenaciously attached to contiguous structures, and a portion of the tumor was found to be tightly adherent to the left lateral recess, which was left in situ after a positive irritative response during the neurostimulation of the XII cranial nerve.

**Figure 6:**
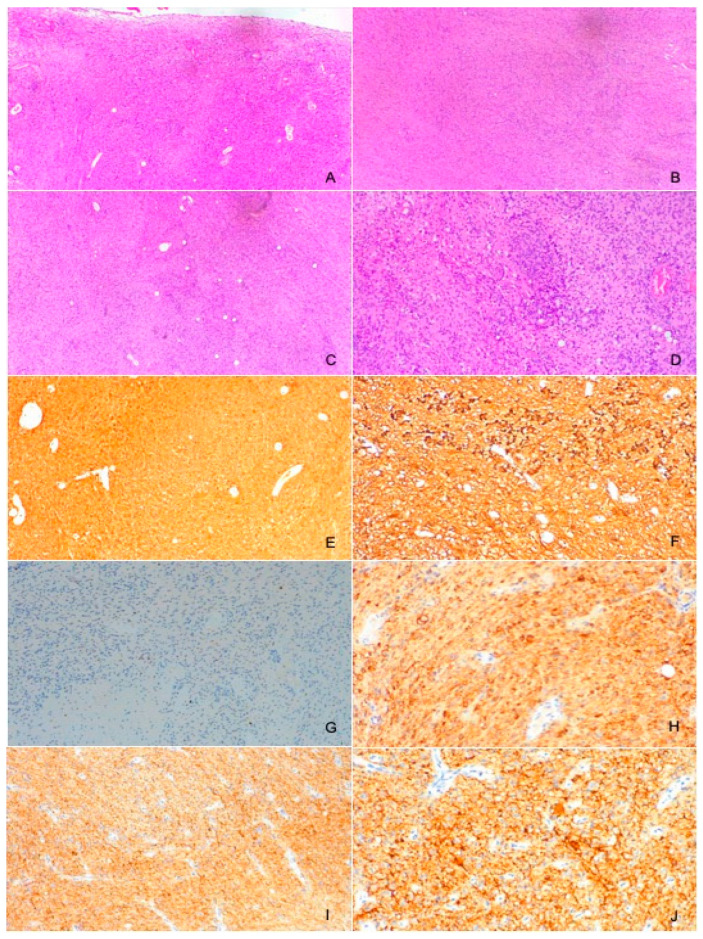
Histopathological analysis revealed fragments diffusely occupied by glial tissue (GFAP+), probably attributable to reactive gliosis (**A**–**F**). Furthermore, the presence of a proliferation was found, consisting in part of glial elements (GFAP+) mixed with neuronal cells (Sinaptophysin+) (**H**–**J**) with a predominantly rounded neurocytic morphology with a preserved nucleus–cytoplasm ratio and very low proliferative index (MIB-1 < 1%) (**G**). No evidence of necrosis or mitosis was identified.

Two years later, a recurrence in the left cerebellar area with inhomogeneous enhancement of 4.5 mm × 15 mm was documented (Figure 4). The patient developed gait disturbances, bilateral left heartbeat nystagmus, a positive Romberg sign, finger-to-nose dysmetria on the left and heel-to-knee dysmetria on the right. A second surgery was performed through a telo-velar approach. The lesion appeared to be a calcified mass tenaciously attached to contiguous structures. A portion of the tumor was found to be tightly adherent to the left lateral recess, which was left in situ after a positive irritative response during the neurostimulation of the XII cranial nerve. The procedure was entirely supported by intraoperative neurophysiological monitoring. The subsequent postoperative course was uneventful (Figure 5). Histopathological examination was consistent with the previous findings of low-grade glioneuronal neoplasia. This entity, not specifically categorized in the current classification (WHO 2021), could be referred to as an infratentorial glioneuronal tumor (Figure 6).

## 4. Discussion

### 4.1. Clinical Presentation

Usually, supratentorial neuroglial neoplasms are located in the temporal lobe [4]. The exclusively intraventricular location of these tumors is rarely observed. MRI findings are nonspecific [33]. Most commonly, the mass presents an iso- or hyposignal on T1-weighted images and an iso- or hypersignal on T2-weighted images. Peripheral gross (“bizarre”) calcifications may be observed. The pattern of the enhancement of the mass by the paramagnetic contrast agent ranges from none to intense [34]. Known to be epileptogenic lesions [4], they are often indicated as long-term epilepsy-associated tumors (LEATs) [2]. The propensity for these tumors to cause seizures may be associated with their location, as well as their abnormal neuronal cells with a potential hyper-excitable state [4]. Patients with an infratentorial tumor location tend to slowly develop symptoms related to intracranial hypertension as a consequence of progressive obstruction of the cerebrospinal fluid (CSF) pathways. Other symptoms such as headache, vomiting, acute neurological deficits and hydrocephalus can be present [2,15]. A faster clinical course can be observed due to acute hydrocephalus or tumor/intraventricular hemorrhage [17].

Contrast-enhanced brain MRI is the gold standard for radiological evaluation. Although some features may guide the diagnosis, such as the classic pattern of a cystic mass with an enhancing mural nodule, the radiological appearance of these oncological entities is strongly heterogeneous, making the pre-operative diagnosis challenging [17].

Some authors proposed the anatomic location of the neoplasm to be the stronger prognostic variable [18]. Although the peculiarities of the series studied herein preclude the drawing of firm conclusions, the last observation may be consistent with cases of infratentorial or ventricular location due to the risk of the obstruction of CSF pathways. In Table 1, the results of our literature review about neuroglial tumor in an adult population with infratentorial location are reported.

### 4.2. Histological Diagnosis

In our illustrative case, the first histopathologic examination reported a non-univocal morphological and immunophenotypic pattern. The diagnosis of a low-grade glioneuronal tumor was then confirmed by a second expert pathologist’s opinion. Considering the lack of information about the diagnosis and management of this tumor, the patient underwent repeated clinical and radiological follow-up. Adjuvant radiotherapy (RT) and chemotherapy after surgery were recommended, although no malignant features were found. To date, there are no previous reports describing infratentorially located low-grade ganglioglioma relapsing after GTR and additional adjuvant therapies.

The cases detected in our literature review underwent a histological evaluation based on different bases coherent with the WHO classification at the time.

In this regard, the classification of the CNS tumor had been based on histological findings for long time until, in the last WHO classification, molecular biomarkers gained importance for providing diagnostic and prognostic information [17]. In the 2016 WHO classification, ganglioglioma was part of the subcategory of “neuronal and mixed neuronal-glial tumors” along with gangliocytomas, rosette forming glioneuronal tumors and others [17]. Molecular parameters were associated with histopathological information correlating with more accurate prognostic parameters for survival and quality of life [17].

### 4.3. Treatment

To date, no established recommendations exist regarding the best treatment for neuroglial tumors in adults at the first diagnosis and recurrence. According to the literature analysis, surgery plays a central role in the treatment of these lesions, especially when gross total resection can be performed. Radio- and chemotherapy represent the most uncharted issues, as there is still no consensus regarding the role of adjuvant treatment. In particular, no clear indications exist about chemotherapy schemes, and no timeframe with the surgical procedure is documented, especially for low-grade lesions. The evaluation is still based on an individual basis and internal multidisciplinary team decisions. The use of post-surgery radiotherapy assumes a slightly more defined role.

#### 4.3.1. Radiotherapy

The effect of radiation on this group of tumors was usually considered to be limited since most are low-grade neoplasms [5]. High-grade lesions, including anaplastic ganglioglioma, liponeurocytoma and some central/extraventricular neurocytomas, may require adjuvant radiation therapy, as supported by conflicting literature findings [19,21]. For example, it has been pointed out that in cases of GTR for anaplastic ganglioglioma, adjuvant radiotherapy may be considered, while in cases of STR, adjuvant chemoradiation therapy should also be considered [22]. Although a large retrospective meta-analysis showed that postoperative RT could significantly improve local control in patients with gangliogliomas who underwent STR, the benefit on overall survival (OS) was not observed [23]. Furthermore, many other studies reported that RT had little impact on progression-free survival (PFS) and OS [5,18,24,25,26]. A recent study by Lin and collaborators [4] showed that adjuvant RT may have a potentially adverse effect on survival in adult patients with low-grade gangliogliomas and that adjuvant chemotherapy has no positive effect on OS in operated patients. They suggested that RT may increase the risk of malignant progression [4]. The literature review revealed that in six cases, the patients underwent radiotherapy. Of these, 50% died from 1 to 12 months after surgery, 16% had a recurrence and 33% had no documented recurrence. STR was performed in 83% of the cases. Malignant change is a rare but well-recognized complication. The transformation of the glial component from a low grade to a higher grade is observed in most cases. Some reports in the literature suggest that radiation may predispose patients to malignant degeneration. The histological types treated with radiotherapy were anaplastic ganglioglioma (33%), ganglioglioma (16%), ganglioblastoma (33%) and xanthoastrocytoma–pleomorphic ganglioma combined (16%). Despite the adjuvant radiotherapy treatment and the low-grade histopathological features, in our case, the recurrence of the tumor occurred two years following surgery.

#### 4.3.2. Chemotherapy

The role of chemotherapy as an adjuvant treatment in neuroglial neoplasms still remains unknown, and it is usually reserved for grade II and III, similarly to radiation therapy [27], although little influence on prognosis was found [5,19,25]. Its potential use may be limited to children in whom radiotherapy is undesirable and patients with discouraging results after surgery and radiotherapy [23,28]. Immunotherapies are spreading nowadays, especially targeting BRAF as an emerging adjuvant therapy in anaplastic ganglioglioma [2,15]. The group of Lundar et al. [24] recently suggested that repeat surgery should be considered before giving adjuvant therapy to patients with incomplete primary resection or recurrent gangliogliomas. Our review found that chemotherapy was performed in only two cases (7%), in a anaplastic ganglioglioma patient subjected to STR who died 10 months after diagnosis and in a ganglioglioma patient. In both cases, temozolamide was given.

#### 4.3.3. Surgical Treatment

Tumor resection is widely accepted as the treatment of choice for these histopathological groups of tumors [11,35]. The surgical approach is related to tumor location. Supratentorial lesions are usually described as well-defined, scarcely hemorrhagic and non-infiltrative neoplasms, while tumor boundaries could be difficult to be defined in most of the ventricular counterparts [8]. Surgical excision should consist of gross total resection (GTR) when possible, since a higher degree of resection (EOR) appears to represent a prognostic factor for these neoplasms. In particular, GTR was associated with OS rates of 100% and 75% for low-grade and high-grade neoplasms, respectively, whereas incomplete resections led to survival rates of 75% and 25%, respectively [8,26]. Although GTR is the best strategy for approaching such lesions, the obsessive pursuit of such an aim could be associated with major neurological deficits. From the data of the review, it emerges that STR was performed in 44% of cases, GTR in 26% of cases and simple biopsy and partial removal in 15% of cases.

Data from the literature about infratentorial gangliogliomas in adults showed that early recurrences can be explained by the higher histological grade (anaplastic ganglioglioma and ganglioblastoma) and the extension of surgical resection. However, our experience does not support such a conclusion since total macroscopic surgical resection was performed and a low-grade ganglioglioma (grade I) was diagnosed. Adjuvant therapies were given according to the oncological consultation provided.

### 4.4. Study Limitations and Future Directions

While this study provides valuable insights into adult infratentorial gangliogliomas, several limitations must be acknowledged. This is a single case presentation and a view of data collected from case reports, and this may limit the generalizability of the results. Future research should aim to address these limitations by recruiting a larger samples and incorporating objective measures where possible, as well as by attempting to lengthen the time of the clinical–radiological observation. Furthermore, this study mainly focused on the radiological, clinical and treatment aspect of the pathology and did not aim to provide concrete definitions. Future investigations could explore the histopathological features related to the type of treatment to provide a more complete understanding of the tumor behavior. Overall, although this study contributes to the existing literature in the collection of experiences regarding adult infratentorial gangliogliomas, its results’ usefulness is limited.

Our case suggests that current knowledge regarding the management of these patients is extremely limited. Accordingly, in the absence of scientific evidence, each patient should be treated on a case-by-case basis with the assistance of a multidisciplinary team in order to offer the best outcome balanced with the best quality of life.

## 5. Conclusions

Infratentorial glioneuronal tumors are rare findings in the adult population. According to the most updated literature review, histopathological characterization does not seem to fully reflect the true behavior of these tumors. Complete surgical resection (GTR) remains the gold standard treatment for the most favorable prognosis when performed safely. Adjuvant therapies represent a supportive treatment but are not yet clearly defined by the firm evidence. Based on the literature data and our experience, each case should be treated on a case-by-case basis with the assistance of a multidisciplinary team. Future studies are needed to properly characterize adult CNS glioneuronal tumors and their histopathological, molecular and genetic behaviors, with the aim of offering a specific prognostic evaluation for each patient.

## Figures and Tables

**Table 1:** Summary of adult patients with a diagnosis of neuroglial tumor with an infratentorial location in the most updated literature search. Abbreviations: Cx—chemotherapy; RT—radiotherapy; N/R—not reported; MRI—magnetic resonance imaging; CT—computed tomography; WI—weighted images.

Author and Year	Age and Sex	Initial Symptoms	Tumor Location	Histological Diagnosis	Surgical Procedure	Cx	RT	Recurrence or Regrowth/Outcome	Imaging Features
Present study, 2024	58, F	Headache, nausea and gait instability	Cerebellum	Ganglioglioma	GTR	No	Yes	Yes/2 y	
Komboz, 2022 [15]	56, M	Asymptomatic (MRI during screening for lung cancer)	Cerebellum	Ganglioglioma	GTR	N/R	N/R	N/R	N/R
Salge, 2021 [9]	25, F	N/R	IV ventricle	Anaplastic ganglioglioma	STR	No	Yes	Yes, 10 months: leptomeningeal dissemination/no further treatment	MRI: iso-hypodense mass filling the fourth ventricle, with a hypodense intralesional area suggesting cystic degeneration/necrosis and contrast enhancement.
Harrison, 2019 [16]	29, M	Headache, dizziness and syncope	IV ventricle	N/R	N/R	N/R	N/R	N/R	N/R
Harrison, 2019 [16]	73, M	N/R	IV ventricle	N/R	N/R	N/R	N/R	N/R	N/R
Bouali, 2018 [11]	40, M	Headache and progressive staggering gait	Cerebellum	Anaplastic ganglioglioma	STR	Yes	Yes	Yes/died 10 months after diagnosis	MRI: hypointense T1-WI and hyperintense T2-WI, peritumoral edema and strong contrast enhancement; CBV: mild peripheral hyperfusion; rCBV = 1.9; MR spectroscopy (MRS): high lactate (1.33) and reduced NAA/creatine ratio.
González Toledo, 2012 [17]	33, M	Right sided weakness and headache	Brainstem	Anaplastic ganglioglioma	Biopsy	N/R	N/R	N/R	MRI: hypointense T1-WI and hyperintense T2 FLAIR WI lesion with a small cystic/necrotic component and partial and irregular contrast enhancement (“patchy pattern”). MRS shows NAA/Cr = 1.37, and it increases in glutamate and choline (Cho = 1.81).
Hsu, 2012 [18]	42, M	N/R	Vermis and IV ventricle	RGNT	N/R	N/R	N/R	No remnant growth	CT: hypodense lesion without contrast-enhancement; MRI: hypointense cystic mass with high signal border on T1-WI and hyperintensity on T2-WI.
Fushimi, 2011 [19]	28, F	Headache	IV ventricle	RGNT	STR	N/R	N/R	No remnant growth	CT: hypodense mass with calcifications; MRI: hypointense T1-WI and hyperintense T2-WI lesion without contrast enhancement.
Matyja, 2011 [20]	20, F	Headache, nausea and balance disturbance	Vermis, partly in the left cerebellar hemisphere and IV ventricle	RGNT	Biopsy	N/R	N/R	No remnant growth	MRI: solid cystic well-demarcated lesion on T1-WI with ring-shaped contrast enhancement.
Arai, 2010 [21]	15, F	Headache	IV ventricle and vermis	RGNT	STR	N/R	N/R	No remnant growth	CT: hypodense lesion without contrast enhancement; MRI: hypointense T1-WI and hyperintense T2-WI lesion without contrast enhancement
Safavi-Abbasi, 2006 [22]	29, M	Longstanding cephalagic episode and worsening headache	Cerebellum with supratentorial extension	Ganglioglioma	STR	N/R	N/R	No remnant growth	MRI: mixed intensity T2-WI with areas with cystic appearance with high signal intensity and areas of solid appearance with low signal intensity.
Mekni, 2006 [13]	25, F	Intracranial hypertension	Cerebellum	Ganglioblastoma	STR	N/R	Yes	Died 1 month after surgery	N/R
Mahlon, 2006 [23]	29, F	Vertigo and headache	IV ventricle	RFGT	STR	N/R	N/R	N/R	MRI: circumscribed heterogeneous mass with cystic areas.
Matzusaki, 2005 [12]	64, F	Dizziness	CPA	Ganglioblastoma	STR	N/R	Yes	Died 1 y after diagnosis	MRI: mixed intensity T1-WI and T2-WI with cystic component and intense contrast enhancement.
Kinoshita, 2002 [24]	28, M	Headache and ataxia	Vermis	Ganglioglioma	STR	N/R	N/R	N/R	N/R
Lagares, 2001 [25]	59, M	Dizziness and syncope	IV ventricle	Gangliocytoma	STR	No	No	No	MRI: hypointense T1-WI and hyperintense T2-WI cystic lesion with intense contrast enhancement.
Lagares, 2001 [25]	19, F	Headache	IV ventricle	Ganglioglioma	GTR	No	No	No	MRI: cystic mass with heterogeneous features and intense contrast enhancement of the nodular component. The cyst wall shows no contrast enhancement.
Lagares, 2001 [25]	36, F	Gait instability and diplopia	IV ventricle	Ganglioglioma	STR	No	No	No	CT: small peripheral dense calcifications.
Evans, 2000 [14]	60, M	Headache and dizziness	Vermis	Xanthoastrocytoma–pleomorphic ganglioglioma combined	STR	No	Yes	No remnant regrowth	N/R
Karamitopoulou, 1995 [26]	38, M	Headache, gait disturbances and hemiparesis	Brainstem	N/R	Partial removal	N/R	N/R	N/R	N/R
Osanai, 1994 [27]	44, M	Hypoventilation and apnea	Superior cerebellar peduncle	N/R	N/R	N/R	N/R	N/R	N/R
Handa, 1994 [28]	53, M	N/R	Cerebellum	N/R	GTR	N/R	N/R	N/R	CT: low-density mass with contrast enhancement. MRI: hyperintense T2-WI mass.
Lindboe, 1992 [29]	27, M	Headache, nausea and ataxia	Cerebellum	N/R	GTR	N/R	N/R	N/R	N/R
Harada, 1998 [30]	22, F	Headache, vomiting and gait disturbances	Cerebellum	N/R	GTR	N/R	N/R	N/R	N/R
Mork, 1979 [31]	22, F	Headache, unsteadiness and slurred speech	Cerebellum	N/R	GTR	N/R	N/R	N/R	N/R
Tommasi, 1966 [32]	23, M	N/R	Brainstem	N/R	Partial removal	N/R	N/R	N/R	CT: hyperdense lesion.
